# Hemodynamic responses to acute angiotensin II infusion are exacerbated in male versus female spontaneously hypertensive rats

**DOI:** 10.14814/phy2.12677

**Published:** 2016-01-12

**Authors:** Ahmed A. Elmarakby, Kanchan Bhatia, Ryan Crislip, Jennifer C. Sullivan

**Affiliations:** ^1^ Departments of Oral BiologyAugusta UniversityAugustaGeorgia; ^2^ Departments of Pharmacology & ToxicologyAugusta UniversityAugustaGeorgia; ^3^ Departments of PhysiologyAugusta UniversityAugustaGeorgia

**Keywords:** Ang II, blood pressure, GFR, sex, SHR

## Abstract

We previously reported that male spontaneously hypertensive rats (SHRs) are more sensitive to chronic angiotensin (Ang) II‐induced hypertension compared with female rats. This study was designed to test the hypothesis that anesthetized male SHRs are also more responsive to acute Ang II‐induced increases in blood pressure and renal hemodynamic changes when compared with female SHRs. Baseline mean arterial pressure (MAP) was higher in male SHRs than in female SHRs (135 ± 2 vs. 124 ± 4 mmHg, *P* < 0.05). Acute intravenous infusion of Ang II (5 ng/kg/min) for 60 minutes significantly increased MAP to 148 ± 2 mmHg in male SHRs (*P* < 0.05) without a significant change in MAP in female SHRs. Baseline glomerular filtration rate (GFR) was also higher in male SHRs than in female SHRs (2.6 ± 0.3 vs. 1.3 ± 0.1 mL/min, *P* < 0.05). Ang II infusion for 60 min significantly decreased GFR in male SHRs (2.0 ± 0.2 mL/min; *P* < 0.05) without significant changes in urine flow rate, sodium, or chloride excretion. In contrast, Ang II infusion increased GFR in female SHRs (1.9 ± 0.2 mL/min; *P* < 0.05). The increase in GFR upon Ang II infusion in female SHRs was associated with increases in urine flow rate (4.3 ± 0.3 to 7.1 ± 0.9 *μ*L/min), sodium excretion (0.16 ± 0.04 to 0.4 ± 0.1 *μ*mol/min), and chloride excretion (0.7 ± 0.08 to 1.1 ± 0.1 *μ*mol/min; for all *P* < 0.05). These findings support the hypothesis that there is sex difference in response to acute Ang II infusion in SHRs with females being less responsive to Ang II‐induced elevations in blood pressure and decreases in GFR relative to male SHRs.

## Introduction

The renin–angiotensin system (RAS) plays a critical role in the regulation of fluid homeostasis and arterial pressure (Fischer et al. 2002; Sandberg and Ji 2003; Brewster and Perazella 2004; Reverte et al. 2013). Under normal physiological conditions, renin is released from granular cells of the juxtaglomerular apparatus in the kidney in response to low blood volume, high salt content in the distal tubules, renal sympathetic nerve activity, and reduced renal perfusion and catalyzes the conversion of angiotensinogen into Ang I. Angiotensin converting enzyme (ACE) then catalyzes the conversion of Ang I into Ang II. Ang II activates angiotensin type 1 (AT‐1) receptors and stimulates aldosterone secretion leading to renal salt and water reabsorption, arteriolar constriction, and subsequent elevations in blood pressure. However, under pathological conditions, over activation of the RAS contributes to the development of hypertension and associated end‐organ damage. Indeed drugs that interfere with the RAS, ACE inhibitors and AT‐1 receptor blockers, are among the most common clinically used antihypertensive drugs (Fischer et al. 2002; Sandberg and Ji 2003; Brewster and Perazella 2004; Reverte et al. 2013).

Sex differences in the RAS have been widely suggested to contribute to sexual dimorphisms in blood pressure and cardiovascular disease; the activity and expression level of numerous components of the RAS are modulated by both the sex chromosome complement of the animal and the gonadal hormone milieu. AT‐1 receptors mediate the prohypertensive actions of Ang II, and renal AT‐1 receptors in particular are critical to the development of Ang II hypertension in male mice (Crowley et al. 2006). In contrast, activation of the “nonclassical RAS pathway” (ACE2, Ang (1–7), angiotensin type 2 (AT‐2), and Mas receptors opposes AT‐1‐mediated effects leading to vasodilation, improved blood flow, and enhanced pressure natriuresis (Sandberg and Ji 2003; Brewster and Perazella 2004; Sullivan et al. 2010a; Crowley and Coffman 2012; Chappell et al. 2014).

Spontaneously hypertensive rats (SHRs) are a genetic model of essential hypertension, and there are well‐established sex differences in the RAS in SHRs (Silva‐Antonialli et al. 2004; Sullivan et al. 2007a, 2007b, 2010a; Sullivan 2008; Maric‐Bilkan and Manigrasso 2012; Xue et al. 2013; Chappell et al. 2014). Male SHRs have greater AT‐1 expression in the kidney, aorta, and mesenteric arteries and exhibit larger increases in blood pressure and renal injury in response to chronic Ang II infusion than female SHRs (Silva‐Antonialli et al. 2004; Yanes et al. 2006; Sullivan et al. 2007a, 2007b; Sullivan 2008). Less is known regarding the acute pressor or renal hemodynamic effects of Ang II in males vs. females; however, hemodynamic responses to Ang II are blunted in women compared with men (Miller et al. 2006; Toering et al. 2015). Sex differences in blood pressure and renal sensitivity to acute Ang II may contribute to sex differences in chronic responses and susceptibility to other cardiovascular diseases. The kidney is critical in the long‐term control of blood pressure, and the highest sensitivity to vascular effects of Ang II is in the kidney. The goal of this study was to test the hypothesis that male SHRs are more sensitive to acute Ang II‐induced increases in blood pressure and renal hemodynamic changes than females.

## Methods

Twelve‐ to thirteen‐week‐old male and female SHRs were used in this study (Harlan Laboratories, Indianapolis, IN). All experiments were conducted in accordance with the National Institutes of Health (*Guide for the Care and Use of Laboratory Animals*) and approved and monitored by the Georgia Regents University Institutional Animal Care and Use Committee. Rats were maintained at the Georgia Regents University animal care facility for 1 week before the study for acclimation purposes. Rats were housed in temperature‐controlled conditions with a 12 h:12 h light–dark cycle. Pilot experiments were performed to determine a dose of Ang II (Phoenix, Burlingame, CA) that produces minimal change in blood pressure in male and female SHRs using graded bolus doses of Ang II (10–50 ng/kg i.v., *n* = 4). Separate sets of male and female SHRs (*n* = 7–8/group) were used to determine the effects of acute Ang II on blood pressure, glomerular filtration rate (GFR), electrolyte excretion, and urine flow rate.

**Figure 1:**
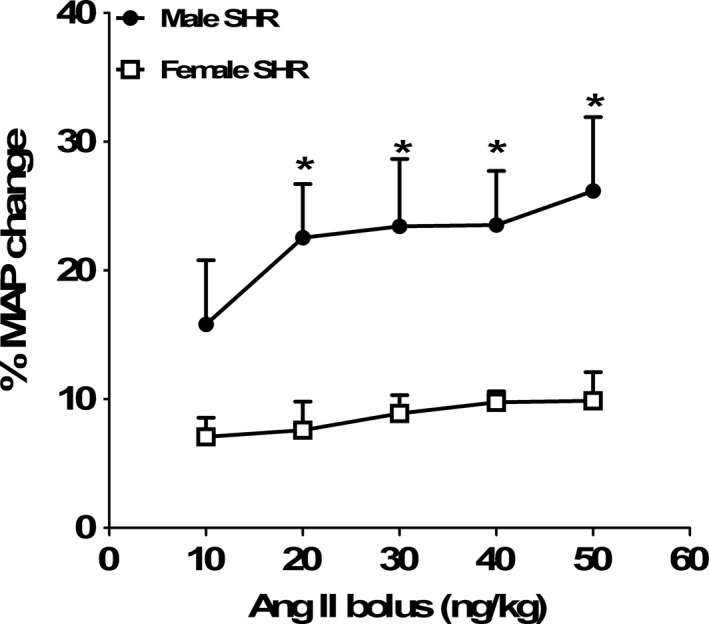
Percentage change in mean arterial blood pressure (MAP) from baseline values in male and female SHRs after bolus injection of graded doses of Ang II (10–50 ng/kg, i.v). Data are mean ± SEM;* n* = 4 rats in each group. *P < 0.05 versus female SHR.

Rats were anesthetized with Inactin (100 mg/kg ip, Sigma, St. Louis, MO) and placed on a servo‐controlled heating table to maintain rectal temperature constant at 37°C. Using polyethylene (PE)‐200 tubing, a tracheostomy was performed to allow unobstructed breathing. A PE catheter (PE‐50) was inserted into the carotid artery for measuring mean arterial pressure (MAP) using a PowerLab data acquisition system (AD Instruments Inc., Colorado Springs, CO). The right jugular vein and femoral vein were cannulated using PE‐50 catheters for continuous infusion of maintenance fluids (6.2% BSA in PBS at 1.8 mL/h) or fluorescein isothiocyanate‐inulin (Sigma, 0.5 mg/kg/min), respectively. Another PE catheter (PE‐90) was placed into the urinary bladder for urine collection. After a 60‐min equilibration period, a 30‐min baseline urine collection period was begun that included a blood sample taken at the midpoint of the period to determine hematocrit values. Following the first 30‐min baseline urine collection, Ang II was infused at a dose of 5 ng/kg/min for 1 h. Based on pilot studies indicating an increase in MAP in male SHRs following a bolus dose of Ang II at 10 ng/kg (Fig. 1), this lower dose was chosen for the continuous infusion. Two additional 30‐min urine collection periods were obtained during Ang II infusion with a blood sample taken at the midpoint for measurement of inulin concentration. Urine volume was measured gravimetrically. Plasma and urinary inulin concentrations were determined using a fluorospectrometer, and values were used to calculate inulin clearance as an indicator of GFR based on the following formula: GFR = (urine flow × urine [inulin])/plasma [inulin]. Urinary sodium, potassium, and chloride concentrations were measured by Medica's electrolyte analyzer (Bedford, MA) to calculate their excretion levels.

Additional anesthetized male and female SHRs (*n* = 5/group) were infused with vehicle (6.2% BSA in PBS at 1.8 mL/h) and fluorescein isothiocyanate‐inulin (Sigma, 0.5 mg/kg/min) to exclude the possibility that changes in hemodynamics in SHRs are due to volume expansion.

### Statistical analysis

All the data are presented as mean ± SEM. Student's *t*‐test for paired data was used to compare MAP changes in response to graded doses of bolus Ang II injection in male and female SHRs (Fig. 1). Evaluation of MAP changes and renal hemodynamic responses to Ang II infusion in male and female SHRs was compared using two‐way ANOVA, factor 1 was sex of the animal, and factor 2 was Ang II treatment. Renal hemodynamic and MAP changes following Ang II infusion were also compared within each sex using repeated‐measures ANOVA**.** Differences were considered statistically significant with *P *<* *0.05. Analyses were performed using GraphPad Prism version 4.0 software (GraphPad Software Inc.)

## Results

Preliminary studies were performed to determine a dose of Ang II that produced a minimal change in arterial pressure in male and female SHRs to exclude changes in blood pressure as a variable in the impact of acute Ang II on renal hemodynamic responses. As shown in Figure 1, bolus injections of graded doses of Ang II (10–50 ng/kg, i.v) produced a dose‐dependent increase in MAP in male SHRs, and this effect was significantly attenuated in female SHRs compared with males. Based on the finding that even the lowest dose of Ang II employed in the pilot study increased MAP in males, subsequent studies were performed using a lower dose of Ang II (5 ng/kg/min).

**Figure 2:**
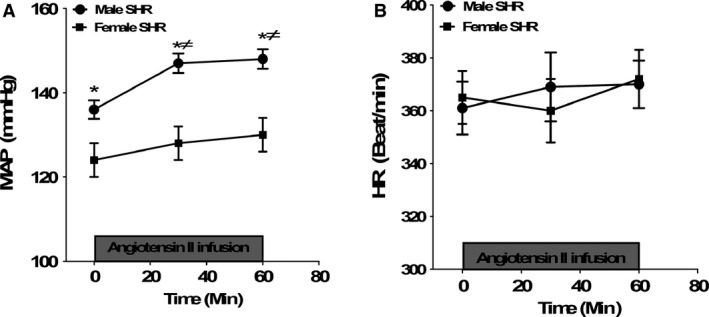
Mean arterial pressure (MAP, panel A) and heart rate (HR, panel B) prior to and during acute Ang II infusion (5.0 ng/kg/min) for 1 h in anesthetized male and female SHR. Data are mean ± SEM;* n* = 7–8 rats in each group. *P < 0.05 versus female SHR; ≠*P* < 0.05 versus baseline.

Figure 2 illustrates MAP and heart rate (HR) before and after 1 h of Ang II infusion in male and female SHRs. Consistent with our previous findings (Reckelhoff et al. 1998; Sullivan et al. 2007a, 2007b, 2010a; Bhatia et al. 2012), baseline MAP was significantly higher in male vs. female SHRs. Infusion of Ang II (5 ng/kg/min, i.v) for 1 h significantly increased MAP in male SHRs (Fig. 2A). MAP in female SHRs was not altered by Ang II infusion (two‐way ANOVA: effect of sex, *P *=* *0.05; effect of Ang II infusion for 30 min, *P *=* *0.001; effect of Ang II infusion for 60 min, *P *=* *0.001; interaction, *P *=* *0.05). There was no difference in HR between male and female SHRs prior to Ang II infusion, and Ang II did not alter HR in either sex (two‐way ANOVA: effect of sex or Ang II infusion, *P *>* *0.05; interaction, *P *=* *0.8, Fig. 2B).

**Figure 3:**
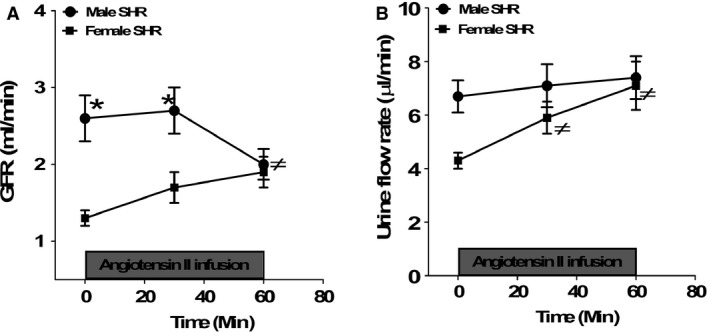
Glomerular filtration rate (GFR, panel A) and urine flow rate (panel B) prior to and during acute Ang II infusion (5.0 ng/kg/min) for 1 h in anesthetized male and female SHR. Data are mean ± SEM;* n* = 7–8 rats in each group. *P < 0.05 versus female SHR; ≠*P* < 0.05 versus baseline.

Baseline GFR was significantly greater in male SHRs than in female SHRs (Fig. 3A). Infusion of Ang II for 1 h decreased GFR in male SHRs. In contrast, GFR was significantly increased by Ang II in female SHRs compared with baseline values (two‐way ANOVA: effect of sex, *P *=* *0.001; effect of Ang II infusion for 30 min, *P *=* *0.01; effect of Ang II infusion for 60 min, *P *=* *0.05; interaction, *P *=* *0.03). Female SHRs also had a lower baseline urine flow rate than males consistent with their lower GFR (Fig. 3B). There was no significant change in urine flow rate after 1 h of Ang II infusion in male SHRs; however, Ang II significantly increased urine flow rate in female SHRs (two‐way ANOVA: effect of sex, *P *=* *0.06; effect of Ang II infusion for 30 min in female SHRs, *P *=* *0.05; effect of Ang II infusion in female SHRs for 60 min, *P *=* *0.05; interaction, *P *=* *0.05).

**Figure 4:**
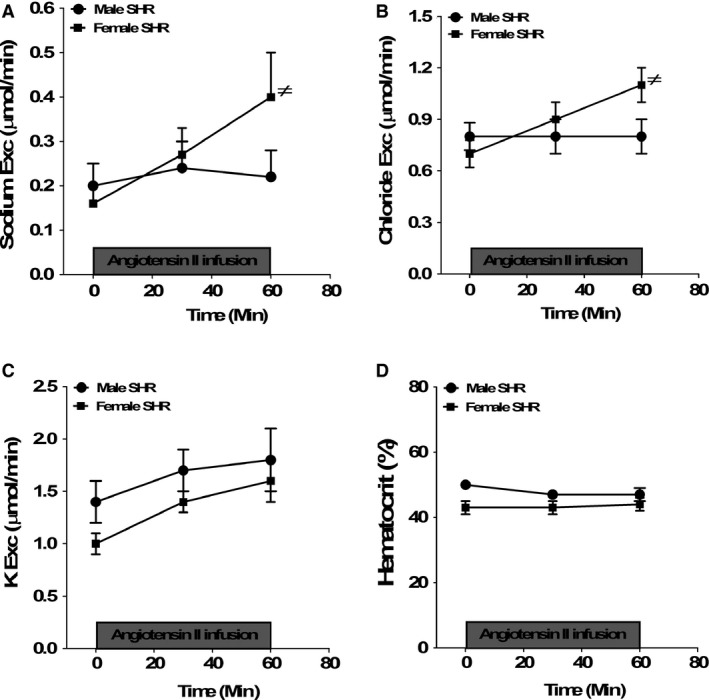
Urinary sodium (panel A), chloride (panel B), and potassium excretion (panel C) levels and hematocrit values (panel D) before and during Ang II infusion for 1 h in anesthetized male and female SHR. Data are mean ± SEM;* n* = 7–8 rats in each group. ≠*P* < 0.05 versus baseline.

Although there were no sex differences in baseline sodium or chloride excretion, infusion of Ang II increased sodium and chloride excretion only in female SHRs (Fig. 4A and B, two‐way ANOVA for sodium excretion: effect of sex, *P *>* *0.05; effect of Ang II infusion for 30 min in female SHRs, *P *>* *0.05; effect of Ang II infusion in female SHRs for 60 min, *P *=* *0.04; interaction, *P *=* *0.1 and two‐way ANOVA for chloride excretion: effect of sex, *P *>* *0.05; effect of Ang II infusion for 30 min in female SHRs, *P *>* *0.05; effect of Ang II infusion in female SHRs for 60 min, *P *=* *0.01; interaction, *P *=* *0.1). Neither sex nor Ang II infusion significantly altered potassium excretion or hematocrit values (Fig. 4C and D).

To exclude the possibility that differential renal responses to Ang II infusion between male and female SHRs were due to volume expansion, male and female SHRs were also infused with vehicle (6.2% BSA in PBS at 1.8 mL/h) for 1 h. Infusion of vehicle did not change MAP, GFR, or urine flow rate in male or female SHRs compared with their baseline values (ΔMAP was −1 ± 7 in males vs. −6 ± 4 mmHg in females, ΔGFR was 0.25 ± 0.4 in males vs. 0.003 ± 0.14 *μ*L/min in females, and Δurine flow rate was 0.002 ± 0.001 in males vs. 0.003 ± 0.002 *μ*L/min in females). There were also no significant changes in HR, electrolyte excretion, or hematocrit values in male or female SHRs after vehicle infusion (data are not shown).

## Discussion

Sex differences in the RAS have been widely suggested to contribute to sexual dimorphisms in blood pressure and cardiovascular disease; there is an extensive literature base describing sex differences in the activity and expression level of numerous components of the RAS under baseline conditions and following chronic infusion of Ang II. Much less is known however, regarding the acute effects of Ang II on blood pressure and renal hemodynamic responses in males vs. females. Remaining questions in the field include (1) do sex differences in blood pressure responses to chronic Ang II simply reflect males reaching a higher end blood pressure than females or are there inherent differences in how blood pressure increases following exposure to Ang II; and (2) based on the central role of the kidney in the long‐term control of blood pressure, are there sex differences in renal hemodynamic responses to acute Ang II? The current study extends previous findings by reporting that female SHRs are less sensitive to acute Ang II‐induced changes in MAP and GFR relative to males. Based on the sensitivity of the kidney to Ang II, attenuated increases in renal hemodynamic responses to Ang II infusion in the female likely offer an additional mechanism by which Ang II‐induced hypertension is reduced in females relative to males.

Clinical and experimental studies suggest that males are more sensitive to chronic Ang II‐induced elevations in blood pressure than females (Tatchum‐Talom et al. 2005; Xue et al. 2007; Sampson et al. 2008; Sullivan et al. 2010a; Bhatia et al. 2012). Chronic Ang II infusion exacerbates hypertension and renal injury to a greater extent in male SHRs compared with females (Sandberg and Ji 2003; Yanes et al. 2006; Sullivan et al. 2007a, 2007b, 2010a; Sampson et al. 2008; Bhatia et al. 2012), and the RAS has been implicated in sex differences in blood pressure and proteinuria in SHRs (Gandhi et al. 1998; Reckelhoff 2001; Fischer et al. 2002; McGuire et al. 2007).

Sex differences have been reported to influence renal function and the progression of renal disease (Coggins et al. 1998; Neugarten 2002; Silbiger and Neugarten 2003). Unanesthetized male Munich‐Wistar rats exhibit higher whole‐kidney and single‐nephron GFR, higher plasma flow, and lower renal vascular resistance than females (Munger and Baylis 1988; Remuzzi et al. 1988; Baylis and Corman 1998). Male SHRs have a blunted pressure–natriuresis relationship and greater sodium reabsorption compared with female SHRs (Reckelhoff 2001) which is consistent with male SHRs having a higher blood pressure than females. There are also sex differences in renal injury markers in SHRs; male SHRs have higher albuminuria than females (Sullivan et al. 2007b). Consistent with our previous findings (Sullivan et al. 2007b, 2010b; Sullivan 2008), male SHRs had a higher baseline pressure than females in the current study. However, contrary to previous findings of Reckelhoff et al. (Reckelhoff et al. 1997), baseline GFR was higher in male SHRs than females and GFR significantly decreased after 1 h of Ang II infusion only in male SHRs indicating that females are better able to maintain renal function compared with males. The discrepancy in the GFR results between Reckelhoff el al. and our study could be attributed to assessing GFR using radioactive iothalamate versus fluorescein isothiocyanate‐inulin in our study in addition to factoring GFR for kidney weight in the Reckelhoff et al. study (Reckelhoff 2001). We also postulate that the higher baseline pressure in male SHRs versus females could drive the elevation in glomerular perfusion pressure and GFR than in female SHRs.

Sex differences have been reported in the renal hemodynamic response to acute Ang II (Miller et al. 1999, 2006). Acute Ang II infusion (100 ng/kg/min) in intrauterine growth‐restricted rats decreased GFR in both males and females, although males exhibited a greater decrease compared with females (Ojeda et al. 2013). Previous studies have demonstrated that SHRs are more sensitive to acute Ang II infusion than normotensive rats. For example, in isolated SHR kidneys, Ang II infusion increased renal vascular resistance which in turn increased perfusion pressure and subsequently increased GFR (Steele et al. 1982) to a greater extent compared with normotensive WKY. Similarly, intracerebroventricular administration of Ang II at 1, 5, and 50 pmol resulted in greater increases in renal blood flow, GFR, urine flow rate, and sodium excretion in male SHRs compared with WKY (Jin et al. 1992). These results likely reflect enhanced renal sensitivity to Ang II in SHR, since intrarenal administration of Ang II (1–3 ng/min) reduced renal blood flow, GFR, urine flow rate, and sodium excretion together with increased renal vascular resistance without changing blood pressure in 6‐week male SHRs but not in WKY (Vyas and Jackson 1995).

Although not assessed in the current study, the differential GFR response to Ang II infusion could be attributed to sex differences in the relative changes in afferent and efferent resistance, with constriction proportionally greater in the efferent arteriole, resulting in an increased glomerular pressure and GFR in female SHRs, whereas the decline in GFR in male SHRs could be due to augmented afferent versus efferent arteriolar constrictive response to acute Ang II infusion. Sexual dimorphisms in renal functional responses to Ang II in SHR are also likely related to sex differences in the expression level of RAS components. Male SHRs have higher AT‐1 expression than females, whereas females have higher AT‐2 receptor expression, and greater Ang (1–7) in female SHRs attenuates the hypertensive response to chronic Ang II infusion (Sullivan et al. 2007b, 2010a; Sullivan 2008). Ang II activation of AT‐1 receptors also increases oxidative stress to a greater extent in male SHRs following chronic Ang II infusion compared with females, which could contribute to changes in renal response to acute Ang II (Bhatia et al. 2012). We postulate that greater Ang (1–7) in female SHRs results in Mas receptor stimulation to buffer increases in blood pressure in response to acute Ang II infusion versus male SHRs, while greater AT‐1 receptor expression and oxidative stress exacerbate Ang II effects in male SHRs (Sullivan et al. 2010a; Bhatia et al. 2012). Future studies will examine the relative role of the AT‐1, AT‐2 and Mas receptor activation as well as oxidative stress in modulating the renal hemodynamic effects of acute Ang II infusion in male and female SHRs.

A limitation of the current study is that the dose of Ang II chosen for the constant infusion period was outside the range of Ang II doses used in the pilot study, and bolus injections were used in the pilot rather than constant infusions. A lower dose was chosen to utilize a dose of Ang II that did not result in increases in blood pressure, with the intent of avoiding a confounding effect of blood pressure on renal hemodynamic responses. However, as noted above, even this low dose of Ang II increased blood pressure in male SHRs over the 60‐min infusion period. An alternative approach for the pilot studies would have been to infuse even lower doses of Ang II at steady rates with step‐wise increases. Regardless, our design did allow us to select a dose of Ang II that resulted in sex‐specific effects on blood pressure and renal hemodynamic responses.

## Perspectives

Close to half of hypertension population are women, yet the majority of basic science studies continue to focus on males despite growing evidence that suggests that the pathways regulating cardiovascular and renal function are distinct in males and females. There are conflicting data in the literature regarding whether there is sex difference in response to Ang II infusion clinically. Although Miller et al. (Miller et al. 1999) reported no sex difference in response to Ang II infusion, Gandhi et al. (Gandhi et al. 1998) reported an effect of gender on baroreflex reactivity during Ang II infusion. Toering et al. (Toering et al. 2015) also recently reported that young healthy men had greater increases in blood pressure and decreases in renal blood flow in response to acute Ang II infusion compared with women. Consistent with these most recent findings, the current study indicates that females are resistant to acute Ang II‐induced changes in blood pressure and renal function. In addition, our group recently published that the same sex differences that we observe in Ang II/Ang (1–7) in our rodent models is also found in healthy men and women; women have more circulating Ang (1–7) than men (Sullivan et al. 2015). We also found that Ang (1–7) levels are positively correlated with enhanced endothelial function in women and our studies in experimental animals support a greater role for Ang (1–7) in the chronic control of blood pressure. Additional studies remain to be done to identify the mechanisms by which females limit acute changes in blood pressure, although based on our clinical and basic science studies greater Ang (1–7) is a likely mechanism. Understanding how females respond to acute Ang II challenge could mechanistically provide insight to improve cardiovascular and renal health outcomes in hypertensive individuals.

## Conflict of Interest

None declared.
